# Acute seizure risk in patients with encephalitis: development and validation of clinical prediction models from two independent prospective multicentre cohorts

**DOI:** 10.1136/bmjno-2022-000323

**Published:** 2022-09-05

**Authors:** Greta K Wood, Roshan Babar, Mark A Ellul, Rhys Huw Thomas, Harriet Van Den Tooren, Ava Easton, Kukatharmini Tharmaratnam, Girvan Burnside, Ali M Alam, Hannah Castell, Sarah Boardman, Ceryce Collie, Bethany Facer, Cordelia Dunai, Sylviane Defres, Julia Granerod, David W G Brown, Angela Vincent, Anthony Guy Marson, Sarosh R Irani, Tom Solomon, Benedict D Michael

**Affiliations:** 1 Institute of Infection, Veterinary, and Ecological Science, University of Liverpool Department of Clinical Infection Microbiology and Immunology, Liverpool, UK; 2 NIHR Health Protection Research Unit in Emerging and Zoonotic Infections at University of Liverpool, Liverpool, UK; 3 Department of Neurology, The Walton Centre NHS Foundation Trust, Liverpool, UK; 4 Faculty of Medical Sciences, Newcastle University Translational and Clinical Research Institute, Newcastle upon Tyne, UK; 5 Neurosciences, Royal Victoria Infirmary, Newcastle upon Tyne NHS Foundation Trust, Newcastle upon Tyne, UK; 6 Encephalitis Society, Malton, UK; 7 University of Liverpool Department of Clinical Infection Microbiology and Immunology, Liverpool, UK; 8 Department of Health Data Science, University of Liverpool Faculty of Health and Life Sciences, Liverpool, UK; 9 Institute of Infection, Veterinary, and Ecological Science, NIHR Health Protection Research Unit in Emerging and Zoonotic Infections at University of Liverpool, Liverpool, UK; 10 Tropical and Infectious Diseases Unit, Liverpool University Hospitals NHS Foundation Trust, Liverpool, UK; 11 UK Health Security Agency, London, UK; 12 Virus Reference Department, UK Health Security Agency, London, UK; 13 Nuffield Department of Clinical Neuroscience, University of Oxford, Oxford, UK; 14 Department of Pharmacology and Therapeutics, University of Liverpool Institute of Systems, Molecular and Integrative Biology, Liverpool, UK

**Keywords:** AUTOIMMUNE ENCEPHALITIS, INFECTIOUS DISEASES, CLINICAL NEUROLOGY

## Abstract

**Objective:**

In patients with encephalitis, the development of acute symptomatic seizures is highly variable, but when present is associated with a worse outcome. We aimed to determine the factors associated with seizures in encephalitis and develop a clinical prediction model.

**Methods:**

We analysed 203 patients from 24 English hospitals (2005–2008) (Cohort 1). Outcome measures were seizures prior to and during admission, inpatient seizures and status epilepticus. A binary logistic regression risk model was converted to a clinical score and independently validated on an additional 233 patients from 31 UK hospitals (2013–2016) (Cohort 2).

**Results:**

In Cohort 1, 121 (60%) patients had a seizure including 103 (51%) with inpatient seizures. Admission Glasgow Coma Scale (GCS) ≤8/15 was predictive of subsequent inpatient seizures (OR (95% CI) 5.55 (2.10 to 14.64), p<0.001), including in those without a history of prior seizures at presentation (OR 6.57 (95% CI 1.37 to 31.5), p=0.025).

A clinical model of overall seizure risk identified admission GCS along with aetiology (autoantibody-associated OR 11.99 (95% CI 2.09 to 68.86) and Herpes simplex virus 3.58 (95% CI 1.06 to 12.12)) (area under receiver operating characteristics curve (AUROC) =0.75 (95% CI 0.701 to 0.848), p<0.001). The same model was externally validated in Cohort 2 (AUROC=0.744 (95% CI 0.677 to 0.811), p<0.001). A clinical scoring system for stratifying inpatient seizure risk by decile demonstrated good discrimination using variables available on admission; age, GCS and fever (AUROC=0.716 (95% CI 0.634 to 0.798), p<0.001) and once probable aetiology established (AUROC=0.761 (95% CI 0.6840.839), p<0.001).

**Conclusion:**

Age, GCS, fever and aetiology can effectively stratify acute seizure risk in patients with encephalitis. These findings can support the development of targeted interventions and aid clinical trial design for antiseizure medication prophylaxis.

**WHAT IS ALREADY KNOWN ON THIS TOPIC**

- We searched MEDLINE for research studies published from 2000 to February 2022, in English, that examined the associations of seizures in encephalitis. Four studies focused on specific clinical settings and subgroups, however, we identified no multicentre studies, and none reported the associations with seizures in encephalitis of all aetiologies.

**WHAT THIS STUDY ADDS**

- This examination of patients with encephalitis reflecting the spectrum of aetiologies from two prospective independent multicentre studies, identified that age, Glasgow Coma Scale on admission, presence of fever and aetiology were strongly associated with seizures. Using these limited parameters in a clinical prediction model, we were able to stratify seizure risk.

**HOW THIS STUDY MIGHT AFFECT RESEARCH, PRACTICE OR POLICY**

- The findings can be used to support the development of targeted interventions, such as early specialist care involvement, for patients at highest risk of seizures and to aid the design of clinical trials of antiseizure medication prophylaxis.

## Introduction

Encephalitis is inflammation of the brain parenchyma, caused by infectious or immune-mediated processes, and is associated with significant morbidity and mortality despite antiviral and/or immune therapies.^1^ Globally, 500 000 children and adults are affected each year.^2^ The clinical presentation is variable, but typically includes acute or subacute onset of altered mental state alongside fever, headache, new-onset focal neurological signs and, in some, seizures.^3^ Seizures have particular significance as they are associated with a worse outcome and may well be amenable to prophylaxis.^4^ Although seizures may be a proxy marker of severe encephalitis, there are a number of mechanisms by which they could lead to further brain inflammation and damage, including hypoxia, excitotoxicity and raised intracranial pressure.^5^ However, the incidence of acute symptomatic seizures is highly variable (between 2% and 67%).^5^ Although there is some limited evidence that possible risk factors include the aetiology of encephalitis, younger age and the degree of cortical involvement, our capacity to predict who is at risk of seizures remains very poor.^4 5^ Consequently, there is insufficient evidence to recommend the use of antiseizure medications (ASM) as standard of care as either primary or secondary prophylaxis.^4 6^ Initiation and escalation of ASM is possible in most healthcare settings, and if proven to improve outcome, could be started rapidly as ASM therapy is agnostic to eventual aetiology.

Therefore, if a high-risk group were established, this could be used to stratify patients for future clinical trials of primary and secondary prophylaxis with ASM or, as a minimum, to identify which patients should be managed in settings with adequate capacity to manage this severe complication.^4^ This study aims to establish the factors associated with seizures in encephalitis as well as develop and validate a seizure prediction model of clinical utility for patients presenting with an acute encephalitis syndrome, in accordance with the WHO approach.^7 8^

## Methods

### Cohort 1 (development cohort)

Patients were recruited through the Aetiology Study of Encephalitis Study led by the UK Health Protection Agency (now UK Health Security Agency (UKHSA)) (Cohort 1).^9^ The study prospectively recruited 203 patients with encephalitis from 24 hospitals in England (2005–2008) serving 5 million people (11% of the English population). The study included any person of any age admitted to hospital with encephalitis, full case definition as previously published.^3^ Computerised tomography (CT), MRI and electroencephalogram (EEG) were performed when clinically indicated. Clinical and postmortem samples received enhanced diagnostic testing guided by a multidisciplinary expert panel.

### Cohort 2 (validation cohort)

A second cohort of 233 patients with encephalitis recruited as part of the Understanding and Improving Outcome of Encephalitis in the UK (Enceph-UK) study was used exclusively as a validation cohort for model development (Cohort 2). Enceph-UK prospectively recruited patients from 31 hospitals in England, Wales and Scotland (2013–2016). Patients were eligible if they were 16 years or older and had clinically suspected encephalitis, using the same case definition as Cohort 1.

## Statistical analysis

### Seizure definition

The three outcome measures were (1) seizure occurrence at any time before or during acute admission, referred to as ‘seizures’, (2) the occurrence of seizures during acute admission, referred to as ‘inpatient seizures’ and (3) the occurrence of status epilepticus. Witness description was used to differentiate focal from generalised seizures. Further subclassification, for example, according to International League Against Epilepsy classification, was not feasible. In Cohort 2, the presence of seizures was recorded as a binary outcome under ‘symptoms on admission (or in current illness, up to 8 weeks prior to admission, including prodrome)’, and, therefore, description of subsequent ‘inpatient’ seizures was not possible.

### Data extraction

Data from the first available cerebrospinal fluid (CSF) analysis were extracted. Cut-offs were taken from the UK Standards for Microbiology Investigations: Investigation of CSF.^10^ Glasgow Coma Scale (GCS) was categorised as normal (15/15), mildly impaired (13–14/15), moderately impaired (9–12/15) or severely impaired (3–8/15). Outcomes were recorded according to the Glasgow Outcome Scale (GOS) 6 months after discharge from hospital. Good recovery was defined as GOS=5 and poor outcome was defined as <5, reflecting at least moderate disability.^11^

### Univariate

All univariate analysis was conducted on Cohort 1. Categorical variables were analysed using χ^2^ or Fisher’s exact test. All continuous variables that were non-parametric were analysed using Mann-Whitney U test. Potential confounding variables were considered to be age, sex, ethnicity and treatment. These confounders were re-reviewed after univariate analysis and assessed for effect modification and interaction using binary logistic regression.

## Model development

Clinical prediction modelling was designed to be applicable to routine clinical practice to stratify seizure risk in patients presenting with the clinical features of acute encephalitis syndrome. Binary logistic regression was used to ascertain predictor variables of seizures in Cohort 1 (SPSS V.26). Due to the limited proportion of patients with a clinically indicated EEG and the risk of data availability bias, EEG results were not considered for inclusion. Collinearity was assessed using correlation matrices and one of any two highly correlated variables omitted. The pattern of missing data was reviewed to assess whether data were missing completely at random. Data not missing ‘completely at random’ by Little’s test but deemed to be missing ‘at random’ were imputed using multiple imputation in clinical variables with >5% missing data. Candidate variables were selected by univariate selection (p<0.25) and those identified in the literature. A selection of strongest contributing predictors was made through backward selection based on likelihood ratio. The final binary logistic regression model based on pooled estimates was converted to a provisional clinical scoring system by dividing regression coefficients of each factor by the smallest regression coefficient among the variables to the nearest integer.

### Model development: inpatient seizures

To aid translation to clinical practice, a second binary logistic regression model for inpatient seizure risk was developed using the same approach to represent (1) risk at time point of admission and, (2) with maximal discrimination, and was assessed using receiver operating characteristics (ROC) curve and Hosmer-Lemeshow test (Cohort 1).

### Model validation

Both scoring systems were internally validated using leave-one-out cross-validation performed in R (The R Foundation).^12^ The provisional scoring system for seizures was externally validated on Cohort 2 using ROC curves, Hosmer-Lemeshow test and calibration plot.

## Results

### Description of Cohort 1

The median (IQR) age was 31 (9–55) years, and 109 (54%) were men. The aetiology included 86 (43%) infectious causes, 42 (21%) immune-mediated and 75 (37%) unknown as previously detailed^9^ (table 1). Immune-mediated causes included 23 (11%) with acute disseminated encephalomyelitis (ADEM), 9 (4%) N-methyl-D-aspartate receptor antibodies and 7 (3%) were defined as ‘voltage-gated potassium channel’ (VGKC) antibodies. At the time of recruitment, distinction between subtypes of antibody directed at epitopes of the VGKC were not available.

**Table 1:** Demographic, clinical and investigatory factors associated with seizures in Cohort 1

Demographics and aetiology	All (n=203)	Seizure (n=121)	No seizure (n=82)	PPV	NPV	OR (95% CI)	P value
Age, years, median (IQR)^+^	31 (9–55)	25 (9–50)	39 (11–60)	–	–	–	p=0.051
Age, years^+^							
≤5	37 (18)	27 (22)	10 (12)	73%	43%	2.07 (0.94 to 4.55)	p=0.207
n (%)							
>5 to ≤18	38 (19)	19 (16)	19 (23)	50	38	0.62 (0.30 to 1.26)	
>18 to ≤40	48 (24)	31 (26)	17 (21)	65	42	1.32 (0.67 to 2.58)	
>40 to ≤60	39 (19)	23 (19)	16 (20)	59	40	0.97 (0.47 to 1.97)	
>60	41 (20)	21 (17)	20 (24)	51	38	0.65 (0.32 to 1.30)	
Sex, n (%)							
Male	109 (54)	67 (55)	42 (51)	61	43	1.18 (0.67 to 2.07)	p=0.560
Aetiology, n (%)^+^							
Autoantibody-associated	**16** (**8**)	**14** (**11**)	**2** (**2**)	**88**	**43**	**5.23 (1.16 to 23.7**)	**p=0.003
HSV	**38** (**19**)	**28** (**23**)	**10** (**12**)	**74**	**44**	**2.17 (0.99 to 4.75**)	
Other immune	**26** (**13**)	**10** (**8**)	**16** (**20**)	**38**	**37**	**0.37 (0.16 to 0.87**)	
Other infectious	**48** (**24**)	**23** (**19**)	**25** (**31**)	**48**	**37**	**0.54 (0.28 to 1.03**)	
Unknown	**75** (**37**)	**46** (**38**)	**29** (**35**)	**61**	**41**	**0.89 (0.50 to 1.60**)	
Ethnicity, n (%)							
White	148 (74)	88 (74)	60 (73)	59	40	1.02 (0.54 to 1.92)	p=0.275
Black	26 (13)	13 (11)	13 (16)	50	39	1.57 (0.69 to 3.58)	
Asian	21 (10)	12 (10)	9 (11)	57	40	1.12 (0.45 to 2.80)	
Mixed	5 (3)	5 (4)	0 (0)	100	41	–	
Other	1 (1)	1 (1)	0 (0)	100	41	–	
Immunosuppressed, n (%)	31 (15)	17 (14)	14 (17)	55	40	1.26 (0.58 to 2.72)	p=0.557
HIV positive, n (%)	18 (9)	9 (7)	9 (11)	50	39	0.65 (0.25 to 1.71)	p=0.384
Symptoms							
Median symptom duration, days (IQR, range)^+^	**7 (2–16, 0–261**)	**5 (1–12, 0-261**)	**9 (4–24, 0–199**)	–	–	–	*p=0.012
GCS at presentation, n/total^+^							
≤8	**29/149**	**27/85**	**2/64**	**93%**	**52%**	**24.00 (5.10 to 112.86**)	**p<0.001
9–12	**30/149**	**19/85**	**11/64**	**63%**	**45%**	**3.07 (1.20 to 7.87**)	*p=0.019
13–14	40/149	21/85	19/64	53%	41%	1.97 (0.84 to 4.59)	p=0.118
15 (ref)	50/149	18/85	32/64	36%	32%		
Presenting report, n/total							
Confusion	72/166	45/107	27/59	63%	34%	0.86 (0.45 to 1.63)	p=0.645
Collapse	25/166	14/107	11/59	56%	34%	0.66 (0.28 to 1.56)	p=0.338
Seizure^+^	43/167	43/108	0/59	100%	48%	–	–
Symptoms, n (%)							
Lethargy	**112** (**55**)	**59** (**49**)	**53** (**65**)	**53**	**32**	**0.52 (0.29 to 0.93**)	*p=0.026
Personality/ behavioural change	130 (64)	79 (65)	51 (62)	61	42	1.14 (0.64 to 2.05)	p=0.652
Stiff neck^+^	**46** (**23**)	**21** (**17**)	**25** (**31**)	**46**	**36**	**0.48 (0.27 to 0.93**)	*p=0.028
Headache^+^	120 (59)	65 (54)	55 (67)	54	33	0.57 (0.32 to 1.02)	p=0.058
Irritability	74 (37)	44 (36)	30 (37)	59	40	0.99 (0.55 to 1.77)	p=0.974
Fever^+^	**158** (**78**)	**101** (**84**)	**57** (**70**)	**64**	**56**	**2.21 (1.13 to 4.34**)	*p=0.019
Any focal deficit on neurological examination^+^	**70** (**35**)	**29** (**24**)	**41** (**50**)	**41**	**31**	**0.32 (0.17 to 0.58**)	**p<0.001
Coma	10 (5)	6 (5)	4 (5)	60	40	1.02 (0.28 to 3.72)	p=1.000
Gastrointestinal	98 (48)	56 (46)	42 (51)	57	38	0.82 (0.47 to 1.44)	p=0.490
Respiratory	41 (20)	24 (20)	17 (21)	59	40	0.95 (0.47 to 1.90)	p=0.876
Photophobia	16 (8)	6 (5)	10 (12)	38	39	0.38 (0.13 to 1.08)	p=0.060
Urinary	**21** (**10**)	**7** (**6**)	**14** (**17**)	**33**	**37**	**0.30 (0.12 to 0.78**)	*p=0.010
Investigations and treatment							
MRI, n/total							
Abnormal	127/168	77/99	50/69	61%	46%	1.33 (0.65 to 2.70)	p=0.430
Consistent with encephalitis	106/167	64/98	42/69	60%	44%	1.21 (0.64 to 2.29)	p=0.558
CT, n/total							
Abnormal	79/173	47/104	32/69	59%	39%	0.95 (0.52 to 1.76)	p=0.878
Consistent with encephalitis	64/167	40/102	24/65	63%	40%	1.10 (0.58 to 2.09)	p=0.766
EEG, n/total							
Abnormal	**98/113**	**76/82**	**22/31**	**78%**	**60%**	**5.18 (1.66 to 16.15**)	**p=0.002
Consistent with encephalitis	**94/110**	**73/79**	**21/31**	**78%**	**63%**	**5.79 (1.89 to 17.80**)	**p=0.001
Focal changes	**46/109**	**41/79**	**5/30**	**89%**	**40%**	**5.40 (1.88 to 15.52**)	**p=0.001
CSF, n/total							
Pleocytosis	**146/197**	**79/116**	**67/81**	**54%**	**27%**	**0.45 (0.22 to 0.90**)	*p=0.021
Low glucose	**62/172**	**29/98**	**33/74**	**47%**	**37%**	**0.52 (0.28 to 0.98**)	*p=0.042
High protein	122/182	69/106	53/76	57%	38%	0.81 (0.42 to 1.52)	p=0.511
Antiseizure medication							
Prescribed	42/52	42/46	0/6	–	–	–	–

*Significant at p<0.05. **Significant at p<0.01. ^+^Candidate variable for multivariate model.

Denominator given when missing data.

Bold fonts denote significant findings.

CSF, cerebral spinal fluid; EEG, electroencephalogram; GCS, Glasgow Coma Score; HSV, Herpes simplex virus; NPV, negative predictive value; PPV, positive predictive value.

### Seizures during the acute illness

In Cohort 1, 121 (60%) patients had a seizure during their acute illness and 103 (51%) had a seizure while an inpatient. Of patients with a known presenting report, 43/167 (26%) presented with a history of seizures, which were reported most frequently in patients with autoantibody-associated, 7/14 (50%), and Herpes simplex virus (HSV), 13/29 (45%), aetiologies. A semiotic description of the seizures was available for 73 (36%) patients, of whom 43 (59%) had generalised seizures only, 14 (19%) had focal seizures only and 16 (22%) had both. Four patients had a history of epilepsy, of whom three had a seizure.

**Figure 1:**
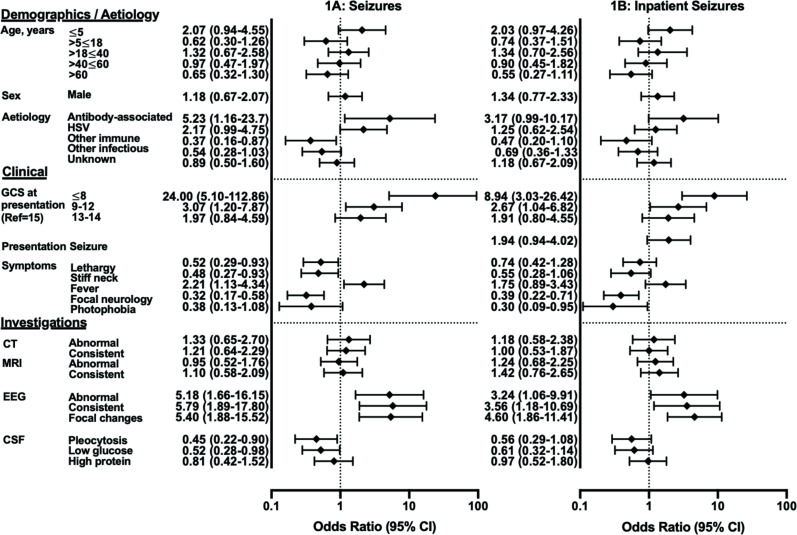
Demographic, clinical and investigatory factors associated with seizures in encephalitis. CSF, cerebrospinal fluid; EEG, electroencephalogram; GCS, Glasgow Coma Scale; HSV, Herpes simplex virus.

Overall, patients with seizures had a lower median (IQR) age at 25 (9–50) years than those without 39 (11–60), (p=0.051) and presented to hospital earlier, at 5 (1–12) versus 9 (4–24) days from symptom onset (p=0.012) (figure 1).

Patients with seizures were less likely to have CSF pleocytosis (OR 0.45 (95% CI 0.22 to 0.90), p=0.021) or low CSF glucose (OR 0.52 (95% CI 0.28 to 0.98), p=0.042).

**Figure 2:**
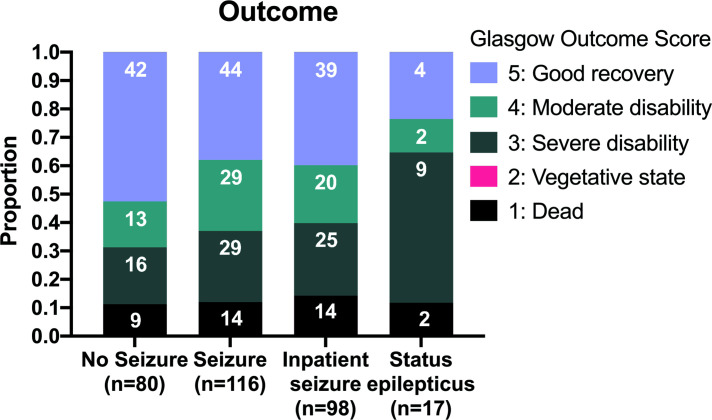
Glasgow Outcome Scale at 6 months stratified by presence and nature of seizures.

Seizures at any point during the acute illness were associated with a worse outcome, with 42/80 (53%) of those with seizures having a poor outcome as opposed to 44/116 (38%) without seizures (OR 1.81 (95% CI 1.01 to 3.23), p=0.044) (figure 2).

### Inpatient seizures

In Cohort 1, inpatient seizures were present in 27/43 (67%) of patients presenting with a history of seizures, and 64/124 (52%) without a history of seizures. In patients who did not present with a history of seizures, those that would go on to develop an inpatient seizure presented with a shorter duration of symptoms at 6 (1–13) versus 9 (4–21) days (p=0.034).

Reduced GCS on admission was associated with subsequent inpatient seizures. This association remained when stratifying data according to whether the patient presented with a history of seizures. In 92 patients with known GCS and without a history of seizures at presentation, those with severely impaired GCS were more likely to have at least one subsequent inpatient seizure (11/13 (85%), OR 6.57 (95% CI 1.37 to 31.5)), compared with those with moderately impaired (11/21 (52%), OR 1.07 (95% CI 0.40 to 2.93)) or mildly impaired/normal GCS (25/58 (43%), OR 0.41 (95% CI 0.17 to 0.99)) (p=0.025).

### Status epilepticus

Status epilepticus occurred in 19/203 (9%) of patients with median (IQR) age of 20 (6–31) years. Three had non-convulsive status epilepticus (NCSE) identified on EEG. Four patients had autoantibody-associated encephalitis, three HSV, two *Mycobacterium tuberculosis*, one probable influenza A, one ADEM and eight had an unknown aetiology. Patients with status epilepticus were more likely to present with a seizure, 10/19 (53%) (of whom three presented with status epilepticus), than those who did not develop status epilepticus 33/148 (22%) (p=0.004). Fever was present in all patients with status epilepticus (19/19, 100%, p=0.009).

All 17 EEGs were abnormal, 16/17 (94%) were consistent with encephalitis and 15/17 (88%) had focal changes. These focal changes were significantly more frequently identified in patients with status epilepticus, 15/17 (88%), compared with those without status epilepticus, 31/92 (34%) (p<0.001).

The probability of subsequent disability was significantly higher in patients with status epilepticus. In survivors with previous status epilepticus a minority, 4/15 (26%) made a good recovery, 2/15 (13%) had mild disability and most 9/15 (60%) had severe disability (figure 2). In survivors without history of status epilepticus most, 82/158 (52%), made a good recovery, 40/158 (25%) had mild disability and 36/158 (23%) severe disability (p=0.028).

### Description of Cohort 2

Patients in Cohort 2 were older, median (IQR), 54 (34–68) years, more likely to be of white ethnicity, 210/233 (91%), and less frequently reported to have a history of seizures, 84/233 (36%), or fever, 102/233 (44%) (online supplemental table 1). Consistent with Cohort 1, autoimmune and HSV aetiology (p=0.002) and low GCS on admission (p<0.001) were associated with seizures.

10.1136/bmjno-2022-000323.supp1 Supplementary data

### Provisional model

Presenting with a seizure and GCS were co-linear, however, GCS was most strongly associated with seizures and likely also captures whether a patient has a history of seizures due to the postictal phase, so was retained in the model. The provisional model of seizures at any time included GCS on admission and probable aetiology of encephalitis (χ2=42.53, p<0.001) (online supplemental table 2). Consistent with the univariate analysis, autoantibody-associated (OR 11.99 (95% CI 2.09 to 68.86), p=0.017) and HSV encephalitis (3.58 (95% CI 1.06 to 12.12), p=0.096) were associated with seizures. Internal cross-validation demonstrated 68% sensitivity, 72% specificity, with a positive predictive value (PPV) of 62% and negative predictive value (NPV) of 77%, with overall accuracy 70%.

**Figure 3:**
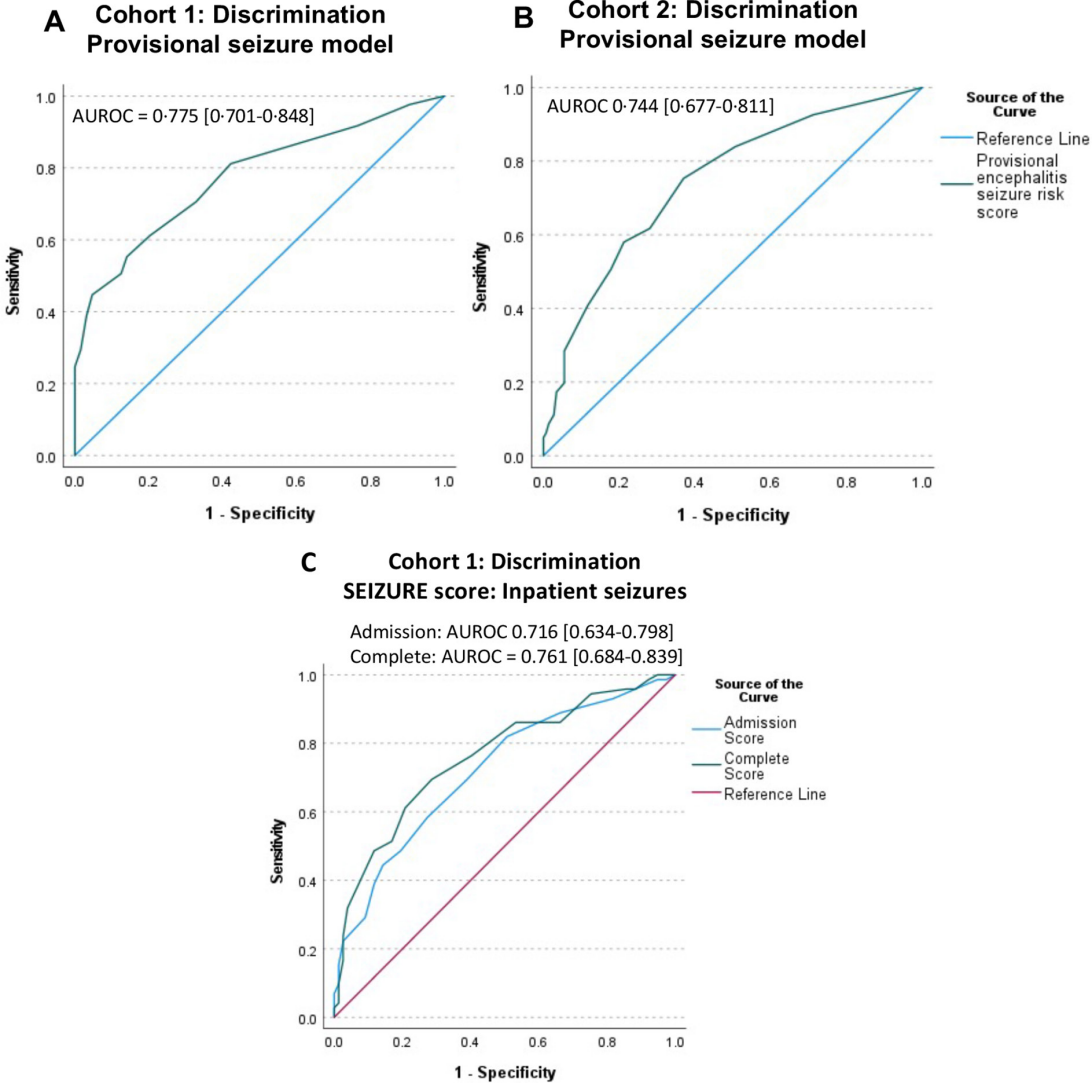
Model performance. Receiver operating characteristics (ROC) curve for seizure risk according to provisional seizure model in Cohort 1 (A), Area under ROC (AUROC)=0.775 (95% CI 0.701 to 0.848), and Cohort 2 (B), AUROC 0.744 (95% CI 0.6770.811). (C) ROC curve for inpatient seizure risk according to SEIZUre Risk in Encephalitis (SEIZURE) score in Cohort 1.

The model demonstrated good discrimination in Cohort 1 and when externally validated in Cohort 2, area under ROC (AUROC)=0.775 (95% CI 0.701 to 0.848) and 0.744 (95% CI 0.677 to 0.811) respectively (figure 3) and Hosmer-Lemeshow test equalled p=0.737 on the original data. Further evaluation of provisional model calibration is provided in online supplemental table 3 and online supplemental figure 1. The provisional model systematically overestimated risk in Cohort 2, but seizures were less commonly reported in Cohort 2 compared with Cohort 1, 84/233 (36%) and 121/203 (60%) patients, respectively.

### Inpatient seizure risk: SEIZURE score

**Figure 4:**
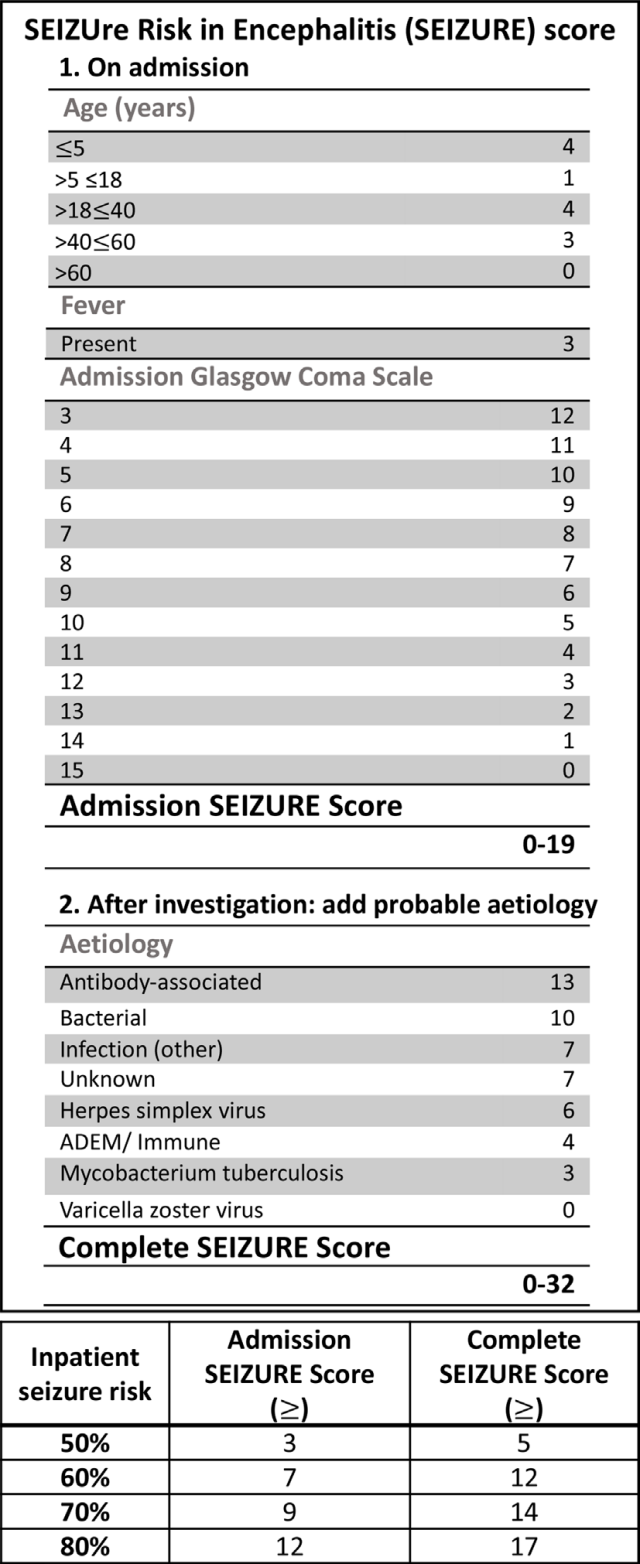
SEIZUre Risk in Encephalitis (SEIZURE) score for stratifying inpatient seizure risk by decile. ADEM, acute disseminated encephalomyelitis.

A second binary logistic regression model was developed to identify predictors of inpatient seizures based on the information available on admission and then on these parameters combined with aetiology once established (Cohort 1) (table 2). The derived, points-based SEIZUre Risk in Encephalitis (SEIZURE) score stratified risk by decile and is designed to be applied by healthcare professionals when a patient of any age with suspected encephalitis is admitted to hospital with two weighted scoring systems for application prior to and following identification of the probable aetiology (figure 4). Internal cross-validation demonstrated 66% sensitivity, 72% specificity, PPV 69% and NPV 69%, with overall accuracy 69%. Patients in the highest risk category on admission had an OR 7.17 (95% CI 2.55 to 20.16) of seizures compared with those in the lowest risk categories and an OR of 15.51 (95% CI 5.60 to 42.96) once probable aetiology was established (table 3).

**Table 2:** Development of scoring system for inpatient seizure risk in encephalitis using binary logistic regression model based on pooled estimates from imputed data in Cohort 1

Inpatient seizure risk: Admission SEIZURE model				
Variable	Model OR (95% CI)	P value	Regression coefficient	Risk score
GCS				
Per point	0.79 (0.69 to 0.90)	0.001	−0.237	1/one point reduction (maximum 12)
Age, years				
≤5	2.37 (0.75 to 7.51)	0.143	0.862	4
>5 to ≤18	1.51 (0.40 to 3.30)	0.792	0.141	1
>18 to ≤40	2.91 (1.07 to 7.90)	0.036	1.068	4
>40 to ≤60	2.17 (0.79 to 5.93)	0.131	0.775	3
>60	1.00	–	–	0
Fever				
Present	2.09 (0.91 to 4.78)	0.082	0.736	3
Model performance: Admission SEIZURE score				Admission score 0–19
AUROC Cohort 1	0.716 (0.634 to 0.798)	<0.001		

Inpatient seizure risk: Aetiology Score				
Variable	Model OR (95% CI)	P value	Regression coefficient	Risk score
Aetiology				
Autoantibody-associated	22.50 (2.50 to 202.93)	0.006	3.113	13
Bacterial	10.03 (1.12 to 89.54)	0.039	2.305	10
Unknown	5.65 (0.88 to 36.22)	0.068	1.728	7
Infection (other)	5.54 (0.66 to 46.53)	0.115	1.711	7
HSV	4.66 (0.69 to 31.77)	0.116	1.540	6
ADEM/immune	2.73 (0.35 to 21.51)	0.340	1.005	4
*Mycobacterium tuberculosis*	2.07 (0.21 to 20.72)	0.536	0.734	3
VZV	1.00	–	–	0
Model performance: Complete SEIZURE score				Complete SEIZURE score 0–32
AUROC Cohort 1	AUROC 0.761 (0.684 to 0.839)	<0.001		

ADEM, acute disseminated encephalomyelitis; AUROC, area under the receivers operating curve; GCS, Glasgow Coma Scale; HSV, Herpes simplex virus; SEIZURE, SEIZUre Risk in Encephalitis; VZV, Varicella zoster virus.

**Table 3:** Performance of SEIZURE score by cut-off in complete data, Cohort 1

Admission SEIZURE score (prior to known aetiology)				
	Inpatient seizure (72/149)	PPV	OR	P value
0–2 (REF)	1/6	17%	–	–
3–6 (REF)	22/62	36%	–	–
7–8	13/30	43%	1.50 (0.62 to 3.61)	p=0.369
9–11	14/23	61%	3.04 (1.15 to 8.08)	p=0.025
12 or more	22/28	78%	7.17 (2.55 to 20.16)	p<0.001

Complete SEIZURE score (once probable aetiology known)				
	Inpatient seizure (72/149)	PPV	OR	P value
0–4 (REF)	0/4	0%	–	–
5–11 (REF)	11/46	24%	–	–
12–13	12/27	44%	2.84 (1.03 to 7.8)	p=0.043
14–16	14/29	48%	3.31 (1.23 to 8.90)	p=0.018
17 or more	35/43	82%	15.51 (5.60 to 42.96)	p<0.001

PPV, positive predictive value; SEIZURE, SEIZUre Risk in Encephalitis.

The admission SEIZURE score, including age, admission GCS and fever, showed good discrimination (AUROC=0.716 (95% CI 0.634 to 0.798)). Addition of probable aetiology, once known, slightly increased discrimination (AUROC 0.761 (95% CI 0.684 to 0.839), p<0.001) and Hosmer-Lemeshow for the complete model was p=0.285 on original data.

## Discussion

Acute seizures affect many patients with encephalitis and are associated with increased need for intensive care support and a worse outcome, and moreover may further contribute to brain injury through excitotoxicity, host immune responses and raised intracranial pressure.^5 12–15^ However, there are currently no established tools to stratify patients as to their risk of seizures and status epilepticus. Without such risk stratification, it is currently not possible to identify which patients would be best managed in centres with adequate clinical facilities, such as those with intensive therapy units and continuous EEG monitoring, and also to identify whom might benefit from primary and secondary ASM prophylaxis.

Through our evaluation of two independent prospective multicentre cohort studies, we identified multiple factors associated with increased risk of seizures during the acute illness, particularly low GCS on admission, fever and an autoantibody-associated or HSV aetiology. Patients with seizures presented to hospital earlier, even in those who have not yet had their first seizure at the time of presentation. Models for seizure risk could be established which, despite requiring a small number of variables, were strongly predictive of acute seizures. Low GCS on admission was more strongly associated with inpatient seizures than whether the patient presented with a seizure history or not. The provisional score accurately determined seizure risk in the first cohort, but potentially overestimated seizure risk in the second cohort, perhaps reflecting under-documentation of seizures in this cohort as seizures were limited to those documented at presentation. The ‘SEIZURE score’ for inpatient seizures requires further external validation. Improved access to easy EEG-monitoring on a wider scale, or the establishment of novel biomarkers could enhance the accuracy of risk-stratification. In addition, the impact of ASM prescription as primary and secondary prophylaxis requires further evaluation.^4 5^

There are likely to be multiple structural and biochemical mechanisms underlying seizure risk in patients with acute encephalitis syndrome. For example, HSV encephalitis has a predisposition to affect epileptogenic areas within the frontotemporal region and in autoimmune encephalitis, the antibodies associated with neuronal cell-surface antigens, that are highly expressed in this region, are themselves often directly involved in the disease process.^16–18^ The differential disease mechanisms observed in specific aetiologies of encephalitis may influence seizure risk, however, there was inadequate power in this analysis to establish factors associated with seizures within aetiological subgroups. Certain clinical features were less common in patients with seizures, specifically, stiff neck, photophobia, lethargy and any focal deficit on neurological examination. This may in part be explained by aetiological distinctions, as focal neurological deficits were most frequently reported in encephalitis caused by Varicella zoster virus and ADEM, which were least strongly associated with seizures; and particularly the latter which is associated with subcortical white matter lesions as opposed to cortical inflammation which drives seizures.^19^ Many clinical features associated with seizures in our analysis are likely to be proxies for underlying mechanisms, rather than risk factors in themselves. Nevertheless, these features can inform future mechanistic studies, particularly through in vivo models of encephalitis.^20^

EEG abnormalities were strongly associated with clinical seizure activity and EEG also identified three cases of NCSE, which is increasingly recognised in encephalitis, particularly autoimmune aetiologies.^21 22^ Status epilepticus in the context of encephalitis is frequently refractory and has a poor outcome.^23 24^ Patients with seizures were less likely to have CSF pleocytosis or low CSF glucose. Given that lumbar puncture is contraindicated until patients are stabilised following a seizure, we hypothesise that this result could be related in part to delayed lumbar puncture, or also may reflect the increased proportion with autoimmune encephalitis in this group.^19^ These CSF parameters likely reflect aetiological distinctions rather than direct biomarkers of seizures. Nevertheless, these data sets did not provide sufficient granularity to determine the CSF white cell count relative to the time from/before a seizure and this requires further study. No relationship was observed between the presence of normal or abnormal CT or MRI findings and seizure risk. It may be that the imaging variables in this analysis were too crude as they were based on retrospective interpretation of clinically indicated scans performed at multiple sites. Volumetric analysis for research purposes of specific brain regions or structures would be more sensitive.^6 25–27^ The finding may additionally reflect the high incidence of seizures in those with autoimmune encephalitis, who often have normal or near-normal neuroimaging. A single-centre study of 94 patients in China found cortical or hippocampal abnormalities on neuroimaging independently predicted progression to super-refractory status epilepticus.^28^ Notably, the potential associations of seizures identified in our study; aetiology, GCS and younger age, were also reported in a single-centre study in Northern India, despite large differences in the cohort.^26^ The likelihood of a seizure being witnessed may confound associations, for example, younger children in community settings may be more likely to have a seizure witnessed.

Our study corroborates previous work demonstrating an association between seizure activity and poor outcome in encephalitis.^14 29–31^ Although seizures may be a proxy marker of severe disease, there are a number of mechanisms through which seizures could cause further brain damage. Seizures cause significant systemic metabolic and biochemical disturbance including hypoxia, hypoglycaemia, metabolic acidosis as well as direct central nervous system perturbations including glutaminergic activity, raised intracranial pressure and blood–brain barrier permeabilisation, as well as low CSF glucose and high CSF lactate.^32^ A study of 144 patients with Japanese encephalitis presenting to hospital in Vietnam, showed that patients with recent seizures had high CSF lactate:glucose ratios and high CSF opening pressures and that patients with opening pressure >25 cm were more likely to die.^14^ A more recent analysis of CSF biomarkers in HSV encephalitis indicated that acute inflammation may drive subsequent synaptic autoimmunity and proposed a trial of post-acute corticosteroids.^33^ In addition, several inflammatory markers have been associated with a lower GCS, increased oedema and a worse outcome in encephalitis, especially the interleukin-1 family relative to their endogenous antagonists.^13^ It remains unknown whether seizures intervene with the underlying encephalitic process.

Despite the high prevalence and prognostic importance of seizures, the most recent Cochrane review concluded that there is insufficient evidence to support or refute the routine use of antiepileptic drugs for the primary or secondary prevention of seizures in viral encephalitis.^4^ A recent randomised controlled trial of secondary prophylaxis for acute symptomatic seizures in children with encephalitis demonstrated that a 4-week course of ASM was comparable to 12 weeks in terms of the incidence of seizure recurrence.^34^ A rabbit model of HSV-1 encephalitis, untreated with aciclovir, showed that all animals that had a seizure became moribund or died, but that phenobarbital prevented seizures and significantly reduced mortality.^35^ Further questions remain regarding choice and duration of antiepileptic agents.^6^ Any intervention strategy would need to consider the high baseline risk of seizures in patients with encephalitis and the presence of subtle and subclinical seizures including NCSE.^14 21 36^

Our findings reflect two relatively large prospectively recruited cohorts but have limitations, principally due to the retrospective nature of seizure-focused analysis. The observational nature of the study has an intrinsic risk of confounding which we have attempted to address in both the analysis and interpretation of results but there may be a residual impact. Additionally, the data were collected from 2005 to 2016 and seizures were not the primary focus of the initial data collection, potentially resulting in missing data. The diagnosis and management of encephalitis may have changed over this time period, particularly the identification of autoantibodies. These factors are balanced by the substantial sample size for a relatively uncommon condition, the granularity of the UKHSA data and the enhanced diagnostic testing performed.

## Conclusion

These finding indicate that patients with seizures during encephalitis present earlier, but despite this, have a worse outcome, suggesting there may be a window of opportunity for intervention that is currently not being exploited. This study provides a foundation for risk stratification of seizures in encephalitis on clinical grounds alone. Biomarkers and improved access to EEG-monitoring could enhance model accuracy and allow for the development of targeted interventions. The SEIZURE score can be used to aid the design of clinical trials of primary and secondary prophylaxis with ASM.

## Data Availability

Data are available upon reasonable request. The de-identified data that support the findings of this study are available from the corresponding author, for any purpose for which there is ethical approval, immediately following publication and ending in September 2022. Researchers should provide a methodologically sound proposal for approval by the UK Health Security Agency, Virus Reference Department. Data are available alongside study protocol.
