# Supplementary material for: Acute seizure risk in patients with encephalitis: development and validation of clinical prediction models from two independent prospective multicentre cohorts
Source: BMJ Neurol Open. 2022 Sep 5;4(2):e000323. doi: 10.1136/bmjno-2022-000323 (PMC9445799; doi:10.1136/bmjno-2022-000323)
Supplement: Supplementary data [file bmjno-2022-000323supp001.pdf]

Supplemental Material

Supplemental Table 1: Demographic, prediction and outcome variables in cohort 1 and 2, indicating any statistically significant differences between the cohorts and the factors associated with seizures in cohort 2. GCS: Glasgow Coma Score, HIV: Human immunodeficiency virus, HSV: herpes simplex virus. Denominator given when missing data.

		**Cohort 1 (n=203)**	**Cohort 2 (n=233)**	**Cohort 1 vs Cohort 2 p value**	**Cohort 2 Seizures (n=84)**	**Cohort 2 No seizures or not recorded (n=149)**	**Cohort 2 Seizure vs no seizure p value**
Age, years, median (IQR)		31 (9-55)	54 (34-68)	p < 0.001	53 (31-67)	55 (36-69)	p = 0.155
Sex, n (%)	Male	109 (54)	118 (51)	p = 0.525	41 (49)	77 (52)	p = 0.685
Aetiology, n (%)	HSV	38 (19)	65 (28)	p = 0.069	29 (35)	36 (24)	p = 0.002
	Autoimmune	42 (21)	39 (17)		22 (26)	17 (11)	
	Unknown	75 (37)	89 (38)		23 (27)	66 (44)	
	Infection (other)	48 (24)	40 (17)		10 (12)	30 (20)	
Ethnicity, n (%)	White	148 (73)	210 (91)	p < 0.001	76 (91)	134 (91)	p = 0.419
	Black	26 (13)	8 (3)		2 (2)	6 (4)	
	Asian	21 (10)	10 (4)		3 (4)	7 (5)	
	Mixed	5 (3)	2 (1)		2 (2)	0 (0)	
	Other	55 (27)	22 (9)		1 (1)	1 (1)	
HIV, n (%)	Yes	18 (9)	9 (4)	p = 0.056	4 (5)	5 (4)	p = 0.725
GCS categories, n/total	≤8	29/149	25/227	p = 0.012	17/81	8/146	p < 0.001
	9-12	30/149	38/227		19/81	19/146	
	13-14	40/149	95/227		29/81	66/146	
	15	50/149	69/227		16/81	53/146	
Symptoms	Fever	158 (78)	102 (44)	p < 0.001	37 (44)	65 (44)	p = 1.000
	Seizure	121 (60)	84 (36)	p < 0.001	-	-	-

Supplementary table 2: Development of provisional scoring system for seizure risk in encephalitis using binary logistic regression model based upon pooled estimates from imputed data in derivation cohort (cohort 1)· AUROC: area under the receivers operating curve, HSV: Herpes Simplex Virus

**Seizure risk – Provisional model**				
**Variable**	**Model OR (95% CI)**	**p-value**	**Regression co-efficient**	**Risk Score**
**GCS**				
0	1·00	-	0	**0**
Per point	0.73 (0.63-0.84)	<0·001	-0·318	**1 / one point reduction (Maximum 12)**
**Aetiology**				
Antibody-associated	11·99 (2·09-68·86)	0·017	2·484	**8**
HSV	3·58 (1·06-12·12)	0·096	1·274	**4**
Unknown	1·90 (0·67-5·37)	0·085	0·641	**2**
Infection (other)	1·39 (0·47-4·13)	0·071	0·328	**1**
Immune (other)	1·00	-		**0**
**Model performance**				**Total Score 0-20**
AUROC Derivation cohort	0·775 [0·701-0·848]	<0·001		
AUROC Validation cohort	0·744 [0·677-0·811]	<0·001		

Supplementary Table 3: Calibration: Expected/observed proportions for provisional seizure risk score in original data, imputed datasets and validation data.

		Predicted				
**Observed**		No Seizure	Seizure	**E**	**O**	**E/0**
Original data	No seizure	44	20	0.537	0.570	0.941
	Seizure	25	60			
Imputation 1	No seizure	59	23	0.542	0.596	0.909
	Seizure	34	87			
Imputation 2	No seizure	59	23	0.557	0.596	0.934
	Seizure	31	90			
Imputation 3	No seizure	57	25	0.532	0.596	0.893
	Seizure	38	83			
Imputation 4	No seizure	50	32	0.606	0.596	1.017
	Seizure	30	91			
Imputation 5	No seizure	49	33	0.616	0.596	1.033
	Seizure	29	92			
Pooled	No seizure	54.8	32.4	0.596	0.570	1.045
	Seizure	27.2	88.6			
External validation (cohort 2)	No seizure	73	73	0.621	0.357	1.741
	Seizure	13	68			

Supplemental Figure 1: Linear calibration plot for provisional seizure model in validation cohort (cohort 2).

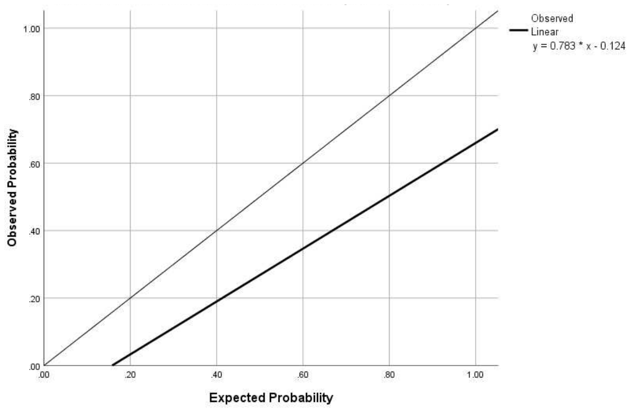

Wood GK, *et al. BMJ Neurol Open* 2022; 4:e000323. doi: 10.1136/bmjno-2022-000323

Regression equations

1) Preliminary model

Log(p/1-p) = 3.523 + 2.484*Aet(Antibody) + 1.274*Aet(HSV) + 0.641*Aet(Unk) + 0.328*Aet(Inf) - 0.318*GCS

2) SEIZURE score

Log(p/1-p)= 0.024 + 0.862*Age(<5) + 0.141*Age(5-18) + 1.068*Age(18-40) + 0.775*Age(40-60) + 0.736*Fever + 3.113*Aet(Antibody) + 2.305*Aet(Bacterial) + 1.731*Aet(Unknown) + 1.711*Aet(Infother) + 1.540*Aet(HSV) + 1.005*Aet(ADEMimm) + 0.728*Aet(MTB) - 0.237*GCS

3) For reference only: Admission SEIZURE score (without aetiology in model)

Log(p/1-p)= 2.048 + 0.601*Age(<5) + 0.173*Age(5-18) + 0.877*Age(18-40) + 0.650*Age(40-60) + 0.466*Fever - 0.244*GCS
